# *c-Myc* Acts as a Competing Endogenous RNA to Sponge *miR-34a*, in the Upregulation of CD44, in Urothelial Carcinoma

**DOI:** 10.3390/cancers11101457

**Published:** 2019-09-28

**Authors:** Pie-Che Chen, Chih-Chia Yu, Wen-Yu Huang, Wan-Hong Huang, Yu-Ming Chuang, Ru-Inn Lin, Jora M. J. Lin, Hon-Yi Lin, Yeong-Chin Jou, Cheng-Huang Shen, Michael W. Y. Chan

**Affiliations:** 1 Department of Urology, Ditmanson Medical Foundation Chiayi Christian Hospital, Chia-Yi 600, Taiwan; chenpiche@gmail.com (P.-C.C.); 01729@cych.org.tw (Y.-C.J.); 2 Department of Radiation Oncology, Dalin Tzu Chi Hospital, Buddhist Tzu Chi Medical Foundation, Chia-Yi 62247, Taiwan; hippo67117@yahoo.com.tw (C.-C.Y.); whitefly@gmail.com (R.-I.L.); doc31221@gmail.com (H.-Y.L.); 3 Department of Biomedical Sciences, National Chung Cheng University, Min Hsiung, Chia-Yi 62102, Taiwancrysital025@gmail.com (W.-H.H.); ccu403250029@gmail.com (Y.-M.C.); a584919@gmail.com (J.M.J.L.); 4 Epigenomics and Human Disease Research Center, National Chung Cheng University, Min Hsiung, Chia-Yi 62102, Taiwan; 5 Department of Health and Nutrition Biotechnology, Asia University, Taichung 41354, Taiwan; 6 Center for Innovative Research on Aging Society (CIRAS), National Chung Cheng University, Min-Hsiung, Chia-Yi 62102, Taiwan; 7 Research Center for Environmental Medicine, Kaohsiung Medical University, Kaohsiung 807, Taiwan

**Keywords:** miR-34a, CD44, c-Myc, ceRNA, urothelial carcinoma

## Abstract

MicroRNAs (miRNAs) have been shown to play a crucial role in the progression of human cancers, including urothelial carcinoma (UC), the sixth-most common cancer in the world. Among them, *miR-34a* has been implicated in the regulation of cancer stem cells (CSCs); however, its role in UC has yet to be fully elucidated. In this study, bioinformatics and experimental analysis confirmed that *miR-34a* targets *CD44* (a CSC surface marker) and *c-Myc* (a well-known cell cycle regulator) in UC. We found that, surprisingly, most UC cell lines and patient samples did express *miR-34a*, although epigenetic silencing by promoter hypermethylation of *miR-34a* expression was observed only in UMUC3 cells, and a subset of patient samples. Importantly, overexpression of *c-Myc*, a frequently amplified oncogene in UC, was shown to upregulate *CD44* expression through a competing endogenous RNA (ceRNA) mechanism, such that overexpression of the *c-Myc* 3′UTR upregulated *CD44*, and vice versa. Importantly, we observed a positive correlation between the expression of *c-Myc* and *CD44* in clinical samples obtained from UC patients. Moreover, overexpression of a dominant-negative p53 mutant downregulated *miR-34a*, but upregulated *c-Myc* and *CD44*, in UC cell lines. Functionally, the ectopic expression of *miR-34a* was shown to significantly suppress *CD44* expression, and subsequently, suppression of cell growth and invasion capability, while also reducing chemoresistance. In conclusion, it appears that aberrant promoter methylation, and *c-Myc*-mediated ceRNA mechanisms, may attenuate the function of *miR-34a*, in UC. The tumor suppressive role of *miR-34a* in controlling CSC phenotypes in UC deserves further investigation.

## 1. Introduction

Urothelial carcinoma (UC) is the sixth most common cancer in the world, with a particularly high incidence in southwestern Taiwan. The majority of UCs are found in the urinary bladder, while tumors from the upper urinary tract (ureter or renal pelvis, known as UTUC) account for 5–10% of all UCs worldwide [1], but 20% in Taiwan [2]. The two principle forms of UC are generally differentiated according to the depth of muscle invasion: non-muscle invasive and muscle invasive cancer. One of the major challenges in treating UC is dealing with the high recurrence rate of superficial cancers, and more than 40% of all UC patients will recur within 5 years, even after surgical treatment [3]. This may be attributed to the presence of drug-resistant cancer stem cells (CSCs), which are characterized by the presence of CD44 [4,5].

CD44 is an integral cell membrane glycoprotein identified as a key biomarker for the isolation and characterization of CSCs [4,6,7]. CD44, a receptor for the extracellular matrix polysaccharide hyaluronate, is involved in several physiological processes—including intercellular adhesion, cell-matrix adhesion, and cell migration—as well as tumor cell invasion and metastasis [8]. Therefore, elucidating the molecular mechanism(s) underlying the formation of CSCs in UC could lead to the development of novel therapeutic methods to deal with this deadly disease.

Epigenetic modification is considered a hallmark of cancer, due to its roles in cellular carcinogenesis and tumor progression [9]. One such modification involves the regulation of microRNAs (miRNAs or miRs), resulting in the generation of small (20–23 nucleotides), non-coding, single-stranded RNAs, which induce the degradation or translational inhibition of homologous target mRNAs by binding their 3′-untranslated region (UTR) [10]. Researchers have also recently identified another mechanism of miRNA regulation, namely, competing endogenous RNAs (ceRNAs), in which an mRNA sharing a common miRNA response element (MRE) with another mRNA, regulate one another by competing for the same miRNA [11]. In this ceRNA mechanism, the 3′UTR of the mRNA could act in trans to modulate gene expression, epigenetically. 

Numerous studies have reported that miRNAs can regulate cancer progression, particularly the processes of growth, invasion, and metastasis [12]. For example, *miR-34a*, which resides on chromosome 1q36.22 and belongs to the *miR-34a* family, has been shown to target the cell cycle regulator, *c-Myc*, in several human cancers [13,14]. In addition, *miR-34a* has also been found to participate in controlling cancer stemness, by targeting CD44 [15,16,17,18].

In this context, *miR-34a* would be considered a tumor suppressor miR in human cancer. Moreover, the promoter region of *miR-34a* contains a CpG island commonly associated with promoter hypermethylation in human cancer [19]. It has also been suggested that *miR-34a* negatively associates with urothelial carcinoma (UC); however, the relationship between the expression of *miR-34a* and its target genes, in UC, has yet to be elucidated. Researchers also have yet to determine the role of epigenetic modification in the expression of *miR-34a*. Consequently, our objective in this study was to determine the role of epigenetic modification in the expression of *miR-34a*, and its target genes, in UC. Unexpectedly, we observed that *miR-34a* is epigenetically controlled by a ceRNA mechanism through the 3′UTR of *c-Myc*, leading to upregulation of *CD44* and cancer progression. 

## 2. Results 

### 2.1. miR-34a Is Epigenetically Silenced in the UMUC3 Cell Line

**Figure 1:**
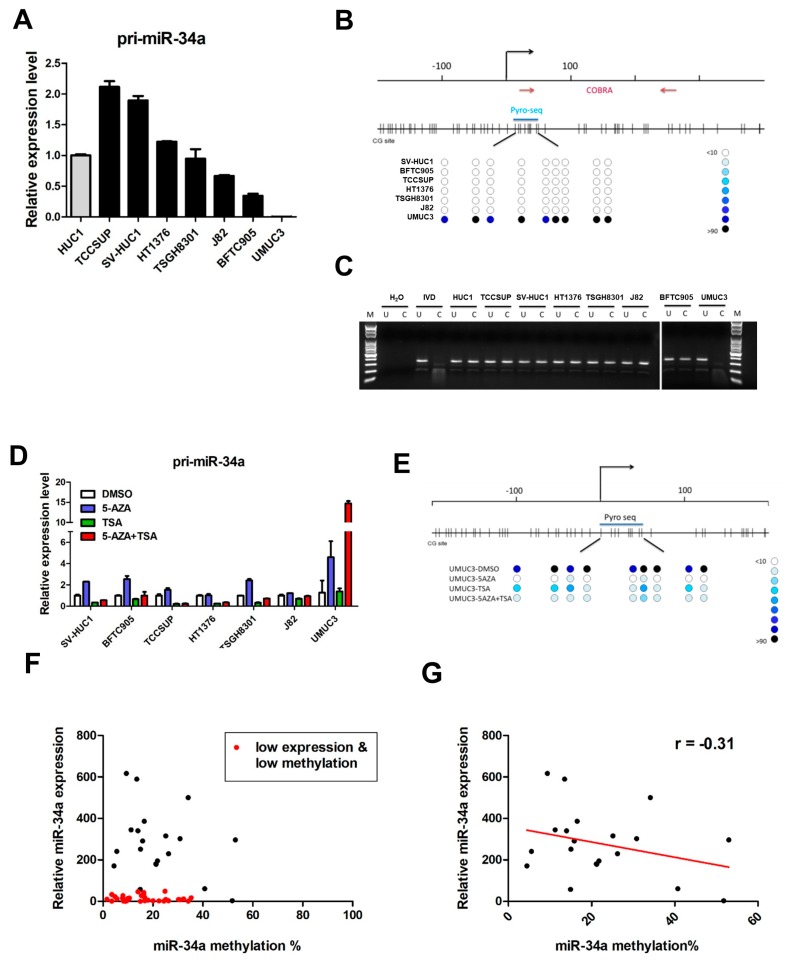
miR-34a is epigenetically silenced by promoter DNA methylation in UMUC3 cell lines, and a subset of urothelial carcinoma (UC) patient samples. (**A**) Expression of pri-*miR-34a* in normal HUC1 bladder cells and various UC cell lines, relative to GAPDH, as an internal control. Methylation analysis of the *miR-34a* promoter in various UC cell lines, using (**B**) bisulphite pyrosequencing and (**C**) COBRA assay. The upper panel in (**B**) presents the genomic structure of the *miR-34a* promoter, with the corresponding locations of all CpG sites from −200 to +400. The lower panel in (**B**) illustrates DNA methylation at each CpG site (circle) of various cells, where the intensity of the blue color indicates the degree of methylation. The CpG sites interrogated by bisulphite prosequencing are also indicated. In the COBRA assays in (**C**), bisulphite-modified DNA was amplified via PCR and digested using *Bst*UI. U, undigested control; C, digested using *Bst*UI; M, DNA ladder marker; *IVD*, in vitro methylated DNA. (**D**) Expression of pri*-miR-34a* in UC cell lines, following epigenetic treatment. UC cell lines treated with 5-aza-2’-deoxycytidine (5aza) and/or trichostatin A (TSA) were examined for pri*-miR-34a* expression, using qRT-PCR. Error bars represent standard deviations calculated from duplicates. (**E**) Bisulphite pyrosequencing analysis of the *miR-34a* promoter in UMUC3 cells treated with various epigenetic drugs, as in (**D**). (**F**) Scatter plot showing expression and promoter methylation of *miR-34a* in 55 urothelial carcinoma patient samples, showing a distinct group of patient samples (red dots) with both low expression and low promoter methylation of *miR-34a*. Analysis of the other patient samples (methylated) showed an inverse correlation (r = −0.31) between expression and methylation of *miR-34a*, as shown in (**G**).

We first examined expression of the immature miR-34a transcript, pri-*miR-34a*, in a panel of normal bladder urothelial cells (HUCs), and seven human UC cell lines. Surprisingly, compared to primary and immortalized (SV-HUC1) HUC cells, there was a dramatic downregulation of *miR-34a* only in UMUC3 cells, while *miR-34a* expression levels varied in the other cell lines (Figure 1A). 

Previous studies have demonstrated that dysregulation of miRNA, through promoter CpG island methylation, is an important mechanism in carcinogenesis [20]. Thus, we sought to identify an epigenetic mechanism downregulating *miR-34a*, in UMUC3 cells. Bisulphite pyrosequencing (Figure 1B), and combined bisulfite restriction analysis (COBRA, Figure 1C) assays were used to determine the methylation status of the CpG island in which *miR-34a* resides. In agreement with the RT-PCR results (Figure 1A), high *miR-34a* methylation levels were observed only in UMUC3 cells. In determining whether DNA methylation was responsible for epigenetic silencing of *miR-34a*, we found that epigenetic treatment with a DNA methyltransferase inhibitor, 5′aza-2′-deoxycytidine (5-AZA), and/or histone deacetylase (HDAC) inhibitor (trichostatin A, TSA), led to robust re-expression of *miR-34a*, in UMUC3 cells only (Figure 1D), due to demethylation of the *miR-34a* promoter region (Figure 1E). These results suggest that *miR-34a* is epigenetically silenced in UMUC3 cells.

Further clinical studies, using 55 UC patient samples, found the *miR-34a* promoter to be devoid of DNA hypermethylation, with only few samples having methylation levels > 50% (Figure 1F). Interestingly, one group of samples (*n* = 35) had low miR-34a expression, regardless of promoter methylation level (Figure 1F, red dot). After exclusion of those samples, the remaining samples (*n* = 20) showed an inverse correlation between miR-34a expression and promoter methylation (Figure 1G, r = −0.31). Together, these studies of UC cell lines and patient samples showed that even lowly expressing miR-34a samples were devoid of high levels of DNA methylation. Thus, an alternative mechanism may be involved in the suppression of *miR-34a* function in UC.

### 2.2. c-Myc Acts as a ceRNA of CD44 in Urothelial Carcinoma

**Figure 2:**
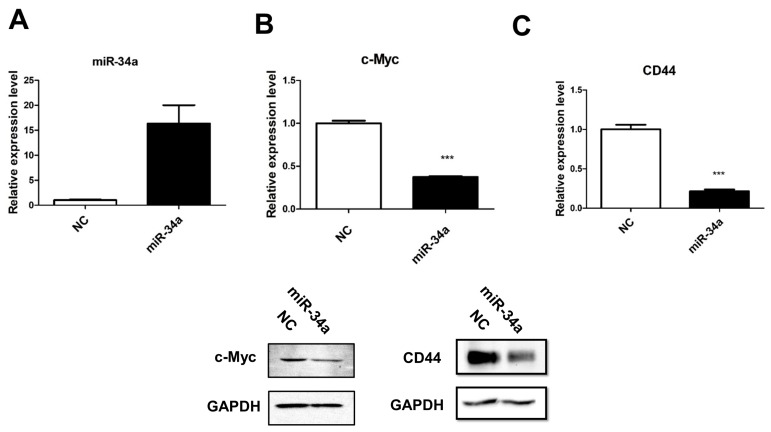
miR-34a targets both *c-Myc* and *CD44*, in UMUC3 urothelial cancer cells. (**A**) Expression of mature miR-34a in UMUC3 cells overexpressing pri-*miR-34a* or an empty vector (NC). Overexpression of miR-34a led to the downregulation of (**B**) c-Myc and (**C**) CD44. Upper panel, RT-PCR; lower panel, western blot analysis. GAPDH was used as a loading control for western blot analysis. (*** *p* < 0.001).

One recent study revealed that mRNAs compete for binding to a shared set of miRNAs, thereby modulating miRNA-based regulation by acting as competing endogenous RNAs (ceRNAs) [21]. We therefore hypothesized that a ceRNA mechanism may be responsible for regulating *miR-34a* in UC. We then conducted a search of the intersection of three databases (TargetScan, miRanda, and PicTar) to identify any *miR-34a* targets overexpressed in UC. Interestingly, numerous studies have reported that *c-Myc,* a known *miR-34a* target [22], is amplified and upregulated in UC [23,24]. To determine whether *miR-34a* targets the *c-Myc* 3′UTR, in UC, we overexpressed *miR-34a* in UMUC3 cells with low *miR-34a* expression (Figure 2A). As expected, transfection of *miR-34a* reduced mRNA and protein levels of *c-Myc* in UMUC3 cells (Figure 2B). Studies have also shown that the stem cell marker, CD44 is a *miR-34a* target in UC [17]. Indeed, overexpression of *miR-34a* was shown to reduce both mRNA and protein of CD44 in UMUC3 cells (Figure 2C). Taken together, these results suggest that *miR-34a* targets *c-Myc* and CD44 in UC.

**Figure 3:**
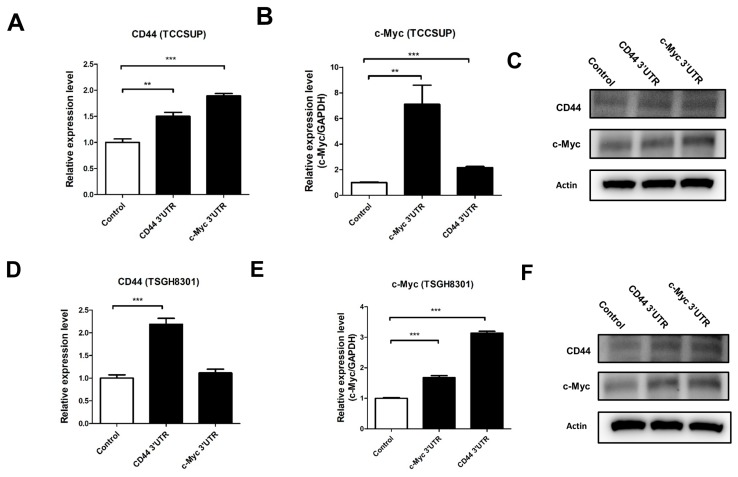
*c-Myc* acts as a ceRNA of *CD44* in urothelial carcinoma cells. TCCSUP (**A**–**C**) or TSGH8301 (**D**–**F**) UC cells overexpressing the *c-Myc* 3′UTR or *CD44* 3′UTR. Expression of CD44 and c-Myc, as evaluated using RT-PCR (mRNA, **A**,**B**,**D**,**E**), and eestern blot analysis (protein, **C**,**F**). GAPDH was used as loading control for western blot analysis. (** *p* < 0.01, *** *p* < 0.001).

**Figure 4:**
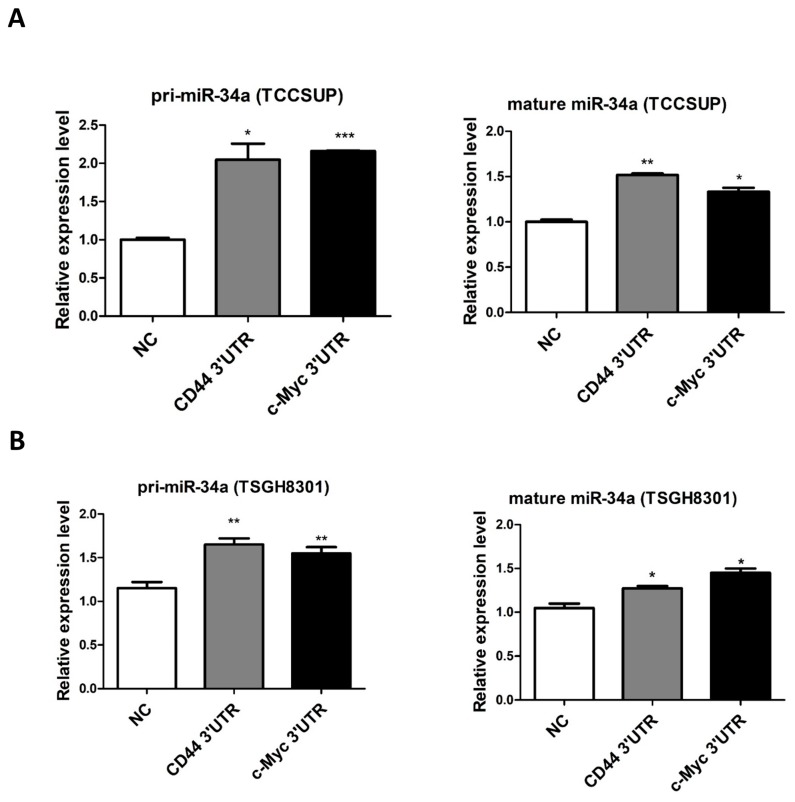
Overexpression of *c-Myc* or *CD44* 3′-UTRs do not suppress *miR-34a* expression in urothelial carcinoma cells. Pri-*miR-34a* and mature *miR-34a* were both detected in (**A**) TCCSUP and (**B**) TSGH8301 urothelial carcinoma cells overexpressing *c-Myc* or *CD44* 3′-UTRs. (* *p* < 0.05; ** *p* < 0.01; *** *p* < 0.001).

The above results indicate that the ectopic expression of *miR-34a* repressed both *c-Myc* and CD44 in UC. Therefore, it was reasonable to assume a significantly positive correlation between *CD44* and *c-Myc* (both targets of *miR-34a* and thus ceRNAs), in UC. To elucidate this ceRNA mechanism, we exogenously overexpressed the *c-Myc* and *CD44* 3′-UTRs, in TCCSUP and TSGH8301 UC cells having high endogenous *miR-34a* expression. Interestingly, overexpression of these 3′-UTRs significantly upregulated the expression of both *CD44* and *c-Myc* mRNA and protein, in TCCSUP (Figure 3A–C) and TSGH8301 (Figure 3D,E) cells. Notably, these events were not due to a decrease, but slight increase, in *miR-34a* expression, both in pre-*miR-34a* and fully mature *miR-34a* (Figure 4A,B). Taken together, these results demonstrate that overexpression of *c-Myc* 3′UTR leads to increased activation of CD44, and vice versa. 

### 2.3. Dicer Knockdown Abolishes ceRNA Effect in UC

**Figure 5:**
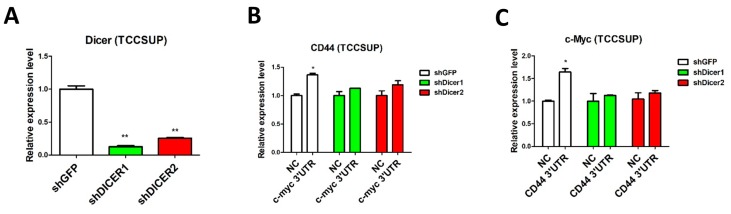

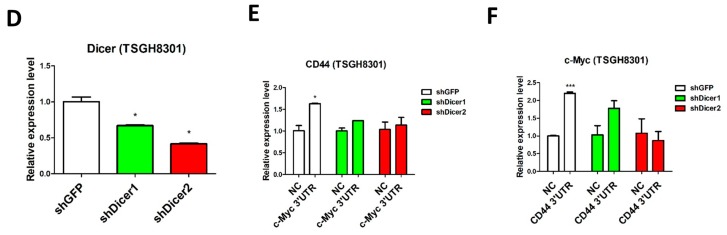
Dicer knockdown diminishes the ceRNA effects of *c-Myc* and *CD44* in urothelial carcinoma cells. Dicer was depleted by shRNA in TCCSUP and TSGH8301 urothelial carcinoma cells. Relative expression of Dicer was assessed in (**A**) TCCSUP and (**D**) TSGH8301 cells using RT-PCR. Expression of *CD44* (**B**,**E**) and *c-Myc* (**C**,**F**) in the control and Dicer-depleted cells was also assessed using RT-PCR. (* *p* < 0.05; ** *p* < 0.01, *** *p* < 0.001).

To determine whether *c-Myc* acts as a ceRNA of CD44 through *miR-34a*, we disrupted miRNA biogenesis via knockdown of the double-strand RNA endoribonuclease, *Dicer* [25], in TCCSUP and TSGH8301 cells. RT-PCR confirmed significant suppression of *Dicer* mRNA expression in both cell types transiently infected with anti-*Dicer* shRNAs (Figure 5A,D). Compared to the negative-control (shGFP), overexpression of the *c-Myc* 3′UTR did not increase *CD44* levels in *Dicer*-depleted TCCSUP (Figure 5B) or TSGH8301 (Figure 5E) cells. Reciprocally, overexpression of the *CD44* 3’UTR did not increase *c-Myc* levels in either Dicer-knocked down cell line (Figure 5C,F). It is also noteworthy that TSGH8301 cells (with lower *Dicer* knockdown efficiency), had increased *c-Myc* and *CD44* expression levels, following overexpression of either 3′UTR, in poorly knocked down *Dicer* cells (Figure 5D–F). Taken together, these results confirm that *c-Myc* and *CD44* act as ceRNAs, through *miR-34a*, in UC.

### 2.4. Expression of c-Myc Correlates with Expression of CD44, in Samples from UC patients and Cell Lines 

**Figure 6:**
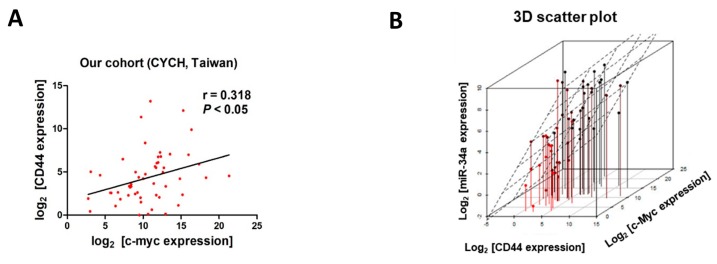

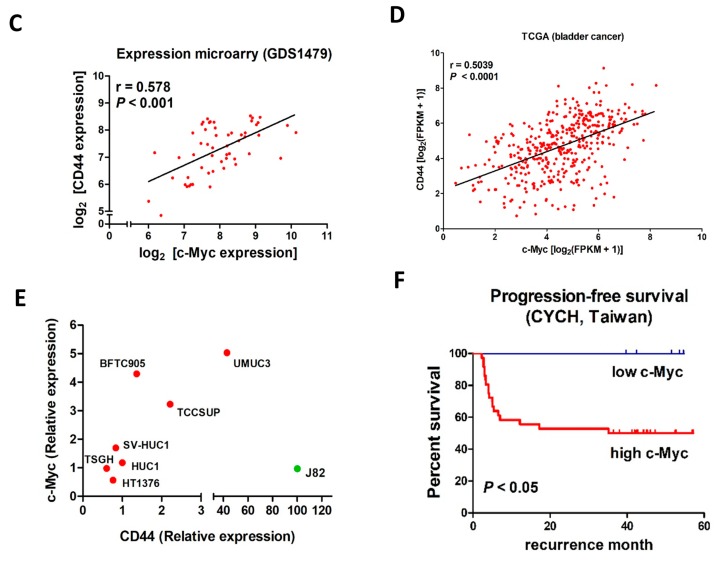
Positive correlation between c-Myc and CD44 expression in samples from urothelial carcinoma patients. (**A**) Scatter plot analysis revealed a positive association between the expression of *c-Myc* and *CD44* (*r* = 0.318, *p* < 0.05) in samples obtained from 55 UC cancer patients at Chia-Yi Christian Hospital (CYCH), Taiwan. (**B**) 3D scatter plot showing positive correlations between *c-Myc*, *CD44*, and *miR-34a* in this cohort of UC patient samples. A positive correlation between the expression of *c-Myc* and *CD44* was also observed in samples obtained from two publicly available databases (**C**) GEO GDS1479 (60 samples, expression microarray, *r* = 0.578, *p* < 0.05) and (**D**) TCGA (411 samples, RNA-Seq, *r* = 0.5039, *p* < 0.001). (**E**) Scatter plot showing the expression *CD44* in UC cell lines. Except for J82 cells (green dot), which showed an exceptionally low expression of *c-Myc*, all other cells showed a positive correlation between expression of these genes (*r* = 0.672). (**F**) Kaplan–Meier survival analysis indicating that higher *c-Myc* expression associates with poor recurrence-free survival (*p* < 0.05).

We also examined the ceRNA mechanism, in vivo, by assessing the expression of *c-Myc* and *CD44* in samples obtained from 55 UC patients. Consistent with our cell line study, the expression of *c-Myc* in the clinical samples positively correlated with the expression of *CD44* (R^2^ = 0.1015, *p* < 0.05, Figure 6A). 3D scatter plot analysis of these samples also revealed a positive correlation between the expression of *miR-34a* and the expression of *c-Myc* and *CD44* (Figure 6B), although no correlation between expression of these genes with any of the clinical-pathological parameters was found (Table 1). Similar findings were obtained from a gene expression array (GDS1479, Figure 6C) and RNA-Seq dataset (from TCGA, Figure 6D). We also investigated the expression of *c-Myc* and *CD44* in HUC1, normal urothelial cells and various UC cell lines (Figure 6E). Except for the J82 cell line, which has surprisingly low levels of *c-Myc* expression (Figure 6E, green dot), all cells demonstrated a positive correlation between *c-Myc* and *CD44* expression, in UC cells (*r* = 0.672). In light of the strong association between c-Myc and the aggressiveness of cancer and eventual prognosis [26], we also examined the correlation between the expression of *c-Myc* and tumor recurrence in this sample cohort. Kaplan–Meier survival analysis revealed an association between higher *c-Myc* expression levels and poor recurrence-free survival (*p* < 0.05, Figure 6F).

### 2.5. Dominant-Negative p53 Mutation Downregulates miR-34a in UC 

**Figure 7:**
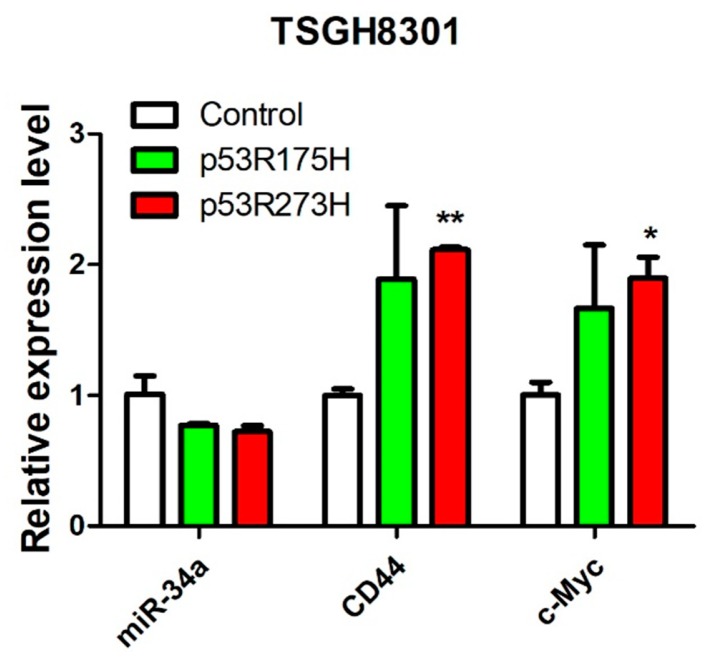
p53 controls *miR-34a* expression in TSGH8301 urothelial carcinoma cells. Co-expression of p53 mutants p53R175H or p53R273H, with wild-type p53, in TSGH8301 cells, downregulated *miR-34a* and upregulated *CD44* and *c-Myc*, as determined by RT-PCR. * *p* < 0.05; ** *p* < 0.01.

Our previous experiment, using clinical samples, found a group of UC patient samples with low *miR-34a* expression, but also low *miR-34a* promoter methylation. This occurrence suggested an alternative mechanism(s) of controlling miR-34a expression in UC. In that regard, the *miR-34a* promoter was found to possess a perfect p53-binding site [27] and be directly regulated by the tumor suppressor p53 [28]. To determine whether p53 could regulate *miR-34a* expression, and the subsequent expression of *c-Myc* and *CD44*, we stably transfected *p53* mutants (*p53*R175H and *p53*R273H) into TSGH8301 UC cells, with endogenous wild-type *p53* [29], to create a dominant-negative effect. Compared to the control, overexpression of the *p53* mutant downregulated *miR-34a* expression, while upregulating *CD44* and *c-Myc* (Figure 7). These results confirmed that p53 regulates *miR-34a* expression directly, and subsequently, miR-34a’s capability of downregulating *CD44* and *c-Myc* expression, in UC cells.

### 2.6. miR-34a Suppresses Tumorigenicity and Drug Resistance in UC

**Figure 8:**
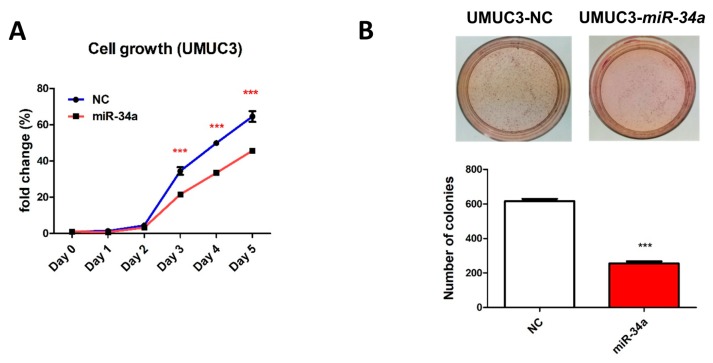

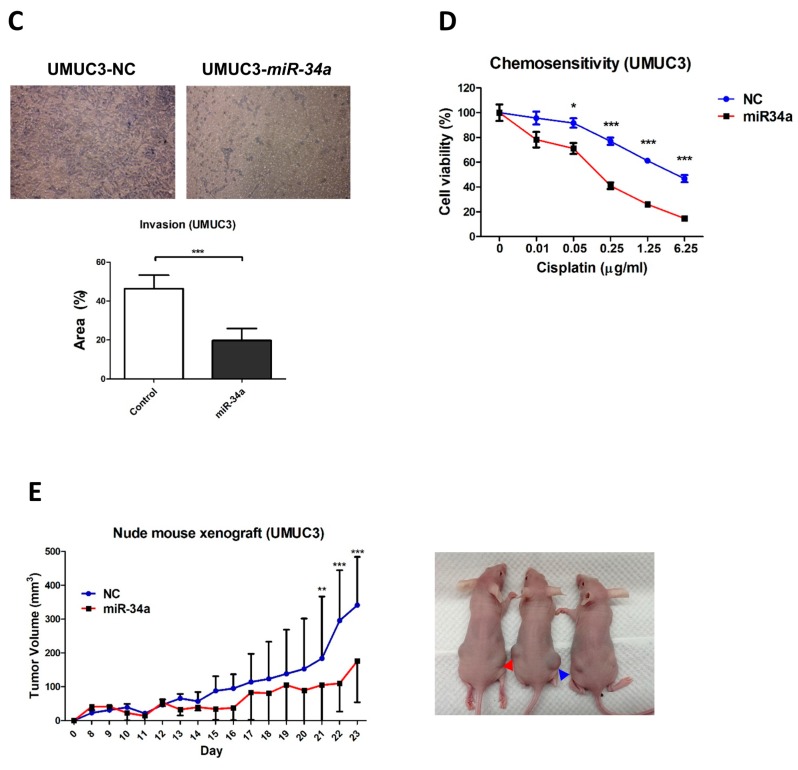
Overexpression of miR-34a suppresses tumorigenicity and drug resistance in UMUC3 UC cells. (**A**) Cell proliferation of UMUC3 cells stably expressing *miR-34a* or an empty vector was measured using cell counting kits (CCKs), on the designated days. Anchorage-independent growth in UMUC3 cells overexpressing *miR-34a* was determined using soft-agar (**B**) and cell invasion (magnification 100×) (**C**) assays. Quantitative analysis of the number of colonies in the soft agar and invasion assays indicated below. (**D**) Chemosensitivity to cisplatin in UMUC3 cells overexpressing miR-34a, as determined using a CCK. (**E**) Nude mouse xenograft model showing tumor sizes of UMUC3 cells overexpressing *miR-34a* (red line) or a control plasmid (blue line). Representative examples of nude mice bearing tumors are also presented (left: miR-34a, red arrow head; right: control, blue arrow head). * *p* < 0.05; ** *p* < 0.01, *** *p* < 0.001.

*miR-34a* has been reported to be a key regulator of tumor suppression, and its downregulation has been linked to the occurrence and development of several cancers [30]. Here, we sought to elucidate the functional role of *miR-34a*, in UC, by stably overexpressing *miR-34a* in UMUC3 cells. Such overexpression significantly inhibited cell growth (Figure 8A), and anchorage-independent growth (Figure 8B). It also significantly inhibited the invasive ability of UMUC3 cells (Figure 8C) and resensitized them to the effects of cisplatin (Figure 8D). Finally, a nude mouse model demonstrated that *miR-34a* overexpression inhibited the growth of UMUC3 cell xenografts in vivo (Figure 8E). Overall, these findings revealed that *miR-34a* overexpression inhibited both the growth and invasive ability of cancer cells, while also reducing chemoresistance in UC cells.

## 3. Discussion

*miR-34a* plays an important role in cancer progression and drug resistance. Previous studies have reported *miR-34a* downregulation in many cancer types [31], including urothelial cancer (UC) [32]. Epigenetic silencing of *miR-34a* has also been addressed in several previous studies of human cancer [19,33,34]; however, the mechanism underlying *miR-34a* downregulation, in UC, has yet to be elucidated. In this study, when evaluating *miR-34a* expression in seven UC cell lines, we were surprised to observe epigenetic silencing of *miR-34a*, via promoter hypermethylation, only in UMUC3 UC cells. Clinical analyses also found that most UC patient samples were devoid of promoter *miR-34a* hypermethylation. For UC cells (BFTC905 and J82) and patient samples showing low expression, but also low *miR-34a* promoter methylation, the possibility of other epigenetic mechanisms (e.g., histone modifications), in regulating *miR-34a*, cannot be excluded. Since *miR-34a* is expressed in most UC cell lines and patient samples, alternative mechanisms likely exist for controlling *miR-34a* function in UC.

A new type of miRNA regulation, referred to as a competitive endogenous RNA (ceRNA) mechanism, involves crosstalk between miRNA targets and the regulation of gene expression, through competition in binding to shared miRNAs has recently been described [35]. Several research groups, including ours, have reported this phenomenon in human cancers [36,37,38,39]. In particular, we recently demonstrated that estrogen receptor-driven upregulation of *E2F6* can ‘sponge’ *miR-193a*, to facilitate *c-Kit* overexpression, in ovarian cancer [36]. In the current study, we demonstrated that overexpression of the *c-Myc* 3′UTR could upregulate *CD44* through *miR-34a*. Importantly, we also observed a positive correlation between the expression of *c-Myc* and *CD44* in samples from UC patients and cell lines. Taken together, our results indicate that *c-Myc* may act as a ceRNA of *CD44*, in UC. 

Several *miR-34a* targets have been shown to control the function of *miR-34a*, through ceRNA mechanisms. For example, one oncogenic, long noncoding (lnc)RNA (lncRNA-SNHG7) has been shown to sponge *miR-34a*, upregulating the expression of the oncogene *GALNT7* in colorectal cancer [40]. In another study, lnc015192 was shown to sponge *miR-34a* to upregulate *Adam12* expression in breast cancer [41]. However, the current study was the first to demonstrate that *c-Myc* mRNA can act as a ceRNA, to upregulate *CD44* expression, by targeting *miR-34a*, in UC. Our results indicate that *c-Myc* participates in cancer stemness, not only via protein regulation [42,43,44], but also via mRNA. Note that the genomic amplification of chromosome 8q (where *c-Myc* resides), commonly associated with UC, provides an additional driving force for the overexpression of *CD44* [23,24]. This may also explain the significantly shorter recurrence-free survival of patients presenting high *c-Myc* expression.

As in previous studies, we observed that *miR-34a* overexpression in UC cells inhibited tumor invasion and reduced drug resistance, probably through the inhibition of CD44 [15,16,17]. In the future, it may be possible to implement epigenetic interventions to restore *miR-34a* expression, and inhibit cancer stemness, in a subset of UC patients with epigenetic silencing of this micro-RNA [16]. 

On the other hand, the tumor suppressor gene *p53*, the most frequently mutated tumor suppressor in human cancer, including urothelial carcinoma [45,46], also directly upregulates *miR-34a* [47,48,49], thereby explaining the fact that p53 signaling can control *miR-34a* expression, in UC samples, without *miR-34a* promoter methylation. In this regard, overexpression of a dominant-negative *p53* mutant resulted in *miR-34a* downregulation, and the subsequent upregulation of *CD44* and *c-Myc*, in UC cells with wild-type p53. 

It is also noteworthy to point out that the ceRNA effect of *c-Myc* and *CD44* overexpression, by the *c-Myc* 3′UTR, in UC cell lines (Figure 4A,B), was not due to a decrease—but surprisingly, a slight increase—of *mR-34a* expression in those cells. This phenomenon may be due to the presence of a feedback loop that increases miR-34a expression upon the increase of *c-Myc*/*CD44* [50]. However, further experiments need to be performed to validate such feedback loop.

## 4. Materials and Methods

### 4.1. Cell Cultures and Patient Samples

Human UC cell lines (SV-HUC, BFTC905, HT1376, TSGH8301, TCCSUP, J82, and UMUC3) were used in this study. SVHUC, BFTC905, HT1376, and TSGH8301 cell lines were maintained in RPMI 1640 medium (Gibco, Grand Island, NY), TCCSUP was maintained in DMEM medium, and J82 and UMUC3 were maintained in MEM medium (Gibco). Cultured cells were supplemented with 10% fetal bovine serum (Gibco) and 1% penicillin-streptomycin, in a humidified atmosphere of 5% CO_2_, at 37 ℃. Fifty-five urothelial cancer samples from bladders or upper urinary tracts (ureter or renal pelvis) were procured from Chia-Yi Christian Hospital, Chia Yi, Taiwan. The clinical-pathological data of all samples is summarized in Table 2. All tissue samples were acquired from either transurethral resection (TURBT) or radical surgery. Patients were then followed up by either cystoscopy or radiographic detection (CT or MRI) for recurrence. All patients provided informed consent. This human sample study was approved by the Institutional Review Board of Chia-Yi Christian hospital, Taiwan.

### 4.2. Plasmid Constructs and shRNA Knockdown

The *miR-34a* full-length DNA sequence was amplified via PCR with specific primers (Table 3) inserted into pSilencer4.1 as miRNA expression vectors. The *CD44* 3′UTR and *c-Myc* 3′UTR regions (each of which contains two *miR-34a* binding sites) were ligated into EGFP-C1. All shRNA plasmids in the pLKO.1 plasmid were purchased from the National RNAi Core Facility at Academia Sinica, Taiwan (rnai.genmed.sinica.edu.tw) and the clone IDs were *Dicer*-1 TRCN0000290426 and *Dicer*-2 TRCN0000290486).

### 4.3. RNA Expression 

All RNA samples were extracted with TRIzol Reagent (Invitrogen, Carlsbad, CA), according to the manufacturer’s instructions. The expression of *CD44* and *c-Myc,* in UC cell lines, was analyzed by RT-PCR, using the StepOne Real-Time PCR System (Applied Biosystems, Waltham, MA, USA) with specific primers (Table 3). Relative gene expression levels were determined by comparing the threshold cycle (i.e., Ct value) of the test gene, normalized to the Ct of GAPDH, in each sample.

### 4.4. DNA Methylation Analysis

All DNA was extracted using Genomic DNA Mini Kits (Geneaid, Taipei, Taiwan) according to the manufacturer’s instructions, and underwent bisulfite modification, using EZ DNA Methylation-Gold™ kits (Zymo Research, Orange, CA, USA), according to the manufacturer’s protocol. Combined Bisulfite Restriction Analysis (COBRA) was used to determine *miR-34a* promoter methylation status, through digestion using *Bst*UI (New England BioLabs, Beverly, MA, USA). Digested products were separated on 1.5% agarose gels for visualization. Pyrosequencing was performed using the PyroMark Q24 (Qiagen, GmbH, Hilden, Germany), with Pyro Gold Reagents (Qiagen), according to the manufacturer’s instructions. The methylation status of nine CpG sites, proximal to *miR-34a*, was measured. The methylation percentage of each cytosine was determined by the fluorescence intensity of a cytosine, divided by the sum of fluorescence intensity of cytosines and thymines, at each CpG site (multiplied by 100%). Primers for methylation analysis are shown in Table 3.

### 4.5. Cell Invasion Assay

2 × 10^4^ cells were seeded in 1% FBS medium in the upper chamber on 2 Millicell™ matrigel-coated (BD Bioscience, NJ, USA) cell culture inserts (Millipore, Billerica, MA). After 48 h, the cells were fixed using methanol and stained using Giemsa reagent (Sigma, St. Louis, MO, USA).

### 4.6. Cell Viability Assay 

Cells were seeded in 96-well plates for 4 d without or with cisplatin in various concentrations. Cell growth was determined using a cell counting kit (CCK-8, Sigma), according to the manufacturer’s instructions. Cell absorbances were then determined at 450 nm, using a microplate reader.

### 4.7. Soft Agar Assay

For soft agar assays, a 3-mL base layer of agar (0.5% agar in culture medium) was allowed to solidify in a six-well flat-bottomed plate, before the addition of 2.0-mL cell suspensions (10,000 cells) in 0.3% agar in culture medium. The cell-containing layer was then solidified at room temperature for 20 min. Colonies were allowed to grow for 14–21 days at 37 °C with 5% CO_2_ before imaging. Plates were then stained with 1 mg/mL iodonitrotetrazolium chloride (Sigma) overnight at 37 °C.

### 4.8. Western Blotting

All samples were lysed using PRO-PREP Protein Extraction Solution (iNtRON Biotechnology, Korea), and then immunoblotted, using monoclonal primary antibodies (CD44, GAPDH, and Dicer), purchased from Santa Cruz Biotechnology (Santa Cruz, CA, USA), and Myc (Abcam, Cambridge, UK). Horseradish peroxidase-conjugated secondary antibodies, including mouse IgG and rabbit IgG antibodies (Abcam), were also used. Specific signals were visualized using a chemiluminescence (ECL) detection kit (Millipore) and the BioSpectrum^®^ 2D Imaging System (BioSpectrum 800, UVP, Upland, CA, USA).

### 4.9. Bioinformatics Analysis 

The output results of multiple prediction programs were integrated using TargetScan (http://www.targetscan.org/), PicTar (http://pictar.org/), and miRanda (http://www.microrna.org/microrna/), to precisely identify miRNAs targeting *c-Myc* and *CD44*. Moreover, results of other microRNAs found in these three databases were retrieved and compared.

### 4.10. In Vivo Tumorigenicity Assay 

Eight-week-old, athymic nude mice (BALB/cByJNarl) were obtained from the Taiwan National Laboratory Animal Center or BioLASCO Taiwan Co., Ltd., Taipei, Taiwan. All mice were kept under specific pathogen-free conditions, using a laminar airflow rack, with free access to sterilized food and autoclaved water. All animal experiments were approved by the Animal Experimentation Ethics Committee of National Chung Cheng University (Taiwan). 1 × 10^6^ cells, in a 1:1 mixture of 0.1 mL medium and Matrigel (BD Biosciences), were injected subcutaneously into each flank of each mouse (day 0). Tumor size was measured daily, using calipers, in length (L) and width (W). Tumor volumes were calculated using the formula (L × W2/2). At the end of the experiments, all mice were sacrificed by cervical dislocation, as previously approved.

### 4.11. Statistical Calculation

All statistical analysis was performed using GraphPad (San Diego, CA, USA) Prism 5 software (version 5.01). Non-parametric variables were assessed using the Mann–Whitney test, when appropriate. Recurrence-free survival was assessed using Kaplan-Meier analysis with log-rank test. A *p* value of less than 0.05 was considered significant.

## 5. Conclusions

In conclusion, *miR-34a* was epigenetically silenced only in a subset of UC cell lines and patient samples. Moreover, overexpression of *c-Myc* was shown to upregulate *CD44*, via a *miR-34a*-mediated ceRNA mechanism, in UC cell lines and clinical samples. The overexpression of *miR-34a* was also shown to suppress tumor growth and invasive capability, while reducing drug resistance. Thus, amplification of *c-Myc* is an important mechanism in controlling cancer stemness, through ceRNA mechanisms, in UC. These novel modes of endogenous competition could explain the regulation of any number of genes, both in healthy and diseased tissues. 

## Figures and Tables

**Table 1:** Correlation between the expression of *miR-34a*, *c-Myc*, *CD44*, and clinical-pathological data in 55 UC samples.

	miR-34a	c-Myc	CD44
**Gender**			
Male	130.2 ± 173.6 ^1^	8.4 ± 4.1	457.3 ± 1.6
Female	31.1 ± 67.5	6.1 ± 1.8	23.8 ± 42.6
**Location**			
Bladder	106.9 ± 157.8	1.7 ± 5.5	512.5 ± 1.7
Upper Tract	105.1 ± 169.1	1.6 ± 6.2	33.8 ± 44.2
**Histological Grade**			
Low Grade	143.7 ± 198.1	2.3 ± 7.6	108.1 ± 270.1
High Grade	100.2 ± 149.8	1.7 ± 5.5	413.5 ± 1.6
**Pathological Stage**			
Stage T1	90.9 ± 149.2	1.7 ± 5.9	68.9 ± 179.1
≥Stage T2	132.2 ± 173.5	1.2 ± 5.3	680.5 ± 2.1
**Relapse**			
Yes	108.6 ± 153.1	1.4 ± 3.4	566.1 ± 1.8
No	102.7 ± 174.4	1.5 ± 5.8	17.9 ± 22.9

^1^ Relative expression value, Mean ± SD.

**Table 2:** Summary of clinical-pathological data of urothelial carcinoma samples.

Clinical Parameter	Patient Samples (*n* = 55)
Age *	70.94 ± 11.8 ^4^
Median	74
Range	40–90
Gender *	
Male	41
Female	13
Location ^1,^*	
Bladder	36
Upper Tract	18
**Histological Grade ^2^**	
Low Grade	12
High Grade	43
**Pathological Stage**	
Stage T1	30
≥ Stage T2	25
Relapse *	
Yes	33
No	21
**Treatment**	
TURBT	34
Non-TURBT ^3^	21

^1^ Upper urinary tract UC from ureter or renal pelvis; ^2^ Grading, low grade: G1, high grade: ≥G2; ^3^ Radial cystectomy (*n* = 2) or nephroureterectomy with bladder cuff excision (*n* = 19); ^4^ Mean ± SD. * 1 case was missing a value.

**Table 3:** Primers used in this study

Primer Name	Primer Sequence (5′ to 3′)	Annealing TEMP (°C)	Product Size (bp)
**COBRA**			
miR-34a-BS-F	TTTTTTTTTTTAGGTGGAGGAG	60	270
miR-34a-BS-R	ATACAAACTTCCAAACCTCTCC		
**Bisulphite Pyrosequeincing**			
miR-34a-Pyro-F	TAGGTGGGGGTTAGGTAG	64	87
miR-34a-Pyro-R ^1^	agctggacatcacctcccacaacgCAAACTCCCACCCCTCCC		
miR-34a-SEQ	GGTAGGGAGTATGAAG		
**RT-PCR**			
pri-miR-34a RT-F	TGTGATTAACCCCGTCTTGCA	60	101
pri-miR-34a RT-R	GCAGATTCTTGAGCCAGATTGC		
CD44-RT-F	TCCCAGTATGACACATATTGC	60	129
CD44-RT-R	CACCTTCTTCGACTGTTGAC		
c-Myc-RT-F	CTCGGATTCTCTGCTCTCTCCTCG	60	177
c-Myc-RT-R	TCTGACCTTTTGCCAGGAGCCT		
Dicer-RT-F	TTAACCTTTTGGTGTTTGATGAGTGT	60	98
Dicer-RT-R	GCGAGGACATGATGGACAATT		
GAPDH RT-F	CCCCTTCATTGACCTCAACTACAT	60	135
GAPDH RT-R	CGCTCCTGGAAGATGGTGA		
**Plasmid construction**			
miR-34a construct			
miR-34a-BamHI F	CGCGGATCCGTAGAGATGGAGTCTTGCTAGTTGC	64	514
miR-34a-HindIII R	CCCAAGCTTTTCTCCCTACGTGCAAACTTCT		
3′UTR construct			
CD44-XhoI F	CCTCGAGTGATCGTTCCAGTTCCCACTTG	66	263
CD44-PstI R	CCCCTGCAGGGGGGTCTGTTGAAGAT		
c-Myc-XhoI F	CCTCGAGCTTGAGACTGAAAGATTTAGCCAT	66	369
c-Myc-PstI R	CCCCTGCAGTAAGATTTGGCTCAATGATATATTTG		

^1^ Primer sequence of the 5′tailed universal primer (UNIVR) is shown as lower case.
